# Solitary metastatic cancer to the thyroid: a report of five cases with fine-needle aspiration cytology

**DOI:** 10.1186/1742-6413-4-5

**Published:** 2007-01-30

**Authors:** Mark W Lee, Yuri K Batoroev, Alexandre N Odashiro, Gia-Khanh Nguyen

**Affiliations:** 1 Department of Laboratory Medicine and Pathology, University of Alberta Hospital, Edmonton, Alberta, Canada; 2 Cytology Laboratory, Irkutsk Oncology Hospital, Irkutsk, Russia; 3 Lac Lab, Campo Grande, Matto Grosso do Sul, Brazil

## Abstract

Three men and 2 women with ages ranging from 37 to 70 years, clinically and histologically confirmed solitary, palpable metastatic cancers to the thyroid (SMCT) and preoperative cytologic investigation of their thyroid lesions by fine-needle aspiration (FNA), were reviewed. Four patients were known to have a solid cancer treated by radical surgery 1 to 4 years prior [1 bronchogenic squamous cell carcinoma, 1 parotid adenoid cystic carcinoma, 1 renal cell carcinoma (RCC) and 1 cutaneous melanoma], and 1 patient had no past history of cancer. Direct smears prepared from the patients' thyroid FNAs were fixed in 95% ethanol and stained with the Papanicolaou method. In 3 cases, immunostaining of the aspirated tumor cells with thyroglobulin antibody was performed, and in 1 case an aspiration smear was stained with commercial HMB-45 antibody. A correct cytodiagnosis of metastatic cancer to the thyroid was made in all 5 cases. In 1 patient the thyroid FNA revealed a metastatic RCC that led to the discovery of a clinically occult RCC. All 5 patients died of metastatic disease 27 to 40 months after surgical resection of their SMCTs.

## Background

Metastatic cancer to the thyroid is found in up to 24% of autopsies of patients who died of disseminated malignant diseases arising from other anatomic sites, and most of those cancer deposits in the thyroid are small and clinically undetectable [1-3]. On rare occasion, a metastatic cancer to the thyroid presents as a solitary and palpable nodule [4,5]. This paper reports five cases of solitary metastatic cancer to the thyroid with challenging fine-needle aspiration (FNA) cytology.

## Patients and methods

Five patients with clinically and histologically confirmed solitary metastatic cancers to the thyroid and preoperative cytologic evaluation by FNA were documented in the files of our 3 laboratories in the past 10 years. The cytologic smears, histologic sections and clinical charts of those patients were reviewed. There were 3 men and 2 women with ages ranging from 37 to 70 years. Four patients had malignant solid cancers resected 1 to 4 years prior and were found to have a palpable solitary thyroid nodule ranging from 3 to 4 cm in greatest dimension during the clinical follow-ups of their malignant diseases. Physical examinations and imaging studies revealed no evidence of metastases in any lymph nodes, organs or recurrent tumor at the sites of their original cancers. The fifth patient had no remarkable past medical history. In all cases, the thyroid nodules were aspirated with a 22-gauge, 4.5-cm-long needle connected to a 10-ml plastic syringe. Direct smears prepared from the needle aspirates were fixed in 95% ethanol and stained by the Papanicolaou technique. In 3 cases, a Papanicolaou-stained cellular smear was stained with a commercial thyroglobulin antibody using the avidin-biotin-complex (ABC) technique without prior destaining with an ethanol-acetic acid solution. In 1 case, an ethanol-fixed cellular smear was stained with a commercial HMB-45 antibody by the same technique. The patients' thyroid lesions were removed by lobectomy. Representative tumor tissue samples from the resected thyroid lobes were processed according to the routine method for histologic study. In one case, 5-micron-thick tissue sections from a representative tumor tissue block were stained with thyroglobulin and HMB-45 antibodies by the ABC technique.

## Results

The clinicopathological data of our 5 patients are tabulated in Table 1. All patients subsequently received chemotherapy and died of metastatic cancer from 27 to 40 months after their thyroid surgeries.

**Table 1:** Clinicopathological Data of Five Patients with Solitary Metastatic Carcinomas to the Thyroid*

**Patient**	**Age/Sex**	**Clinical Data**	**Cytodiagnosis**	**Histodiagnosis**	**Follow-up Data**
1	37/Male	3-cm right STN. Squamous cell carcinoma, upper lobe of right lung removed by lobectomy 8 mos prior	Met. squamous cell carcinoma	Met. squamous cell carcinoma	DWD 27 mos after TS
2	60/Female	3.5-cm left STN Adenoid cystic carcinoma of left parotid removed by radical surgery 2 yrs prior	Met. adenoid cystic carcinoma	Met. adenoid cystic carcinoma	DWD 36 mos after TS
3	48/Male	3-cm right STN RCC of right kidney removed by radical nephrectomy 1 yr prior	Met. RCC, CT	Met. RCC, CT	DWD 38 mos after TS
4	70/Male	4-cm right STN Scalp melanoma treated by wide surgical resection 4 yrs prior	Met. amelanotic melanoma	Met. amelanotic melanoma	DWD 29 mos after TS
5	57/Female	3.4-cm left STN No history of cancer	Met. RCC, CT	Met. RCC, CT	Occult RCC of right kidney detected. DWD 40 mos after TS.

* STN, solitary thyroid nodule; Met, metastatic; DWD, died with metastatic disease; TS, thyroid surgery; RCC, CT, Renal cell carcinoma, clear cell type

### Cytologic and immunocytochemical findings

**Figure 1:**
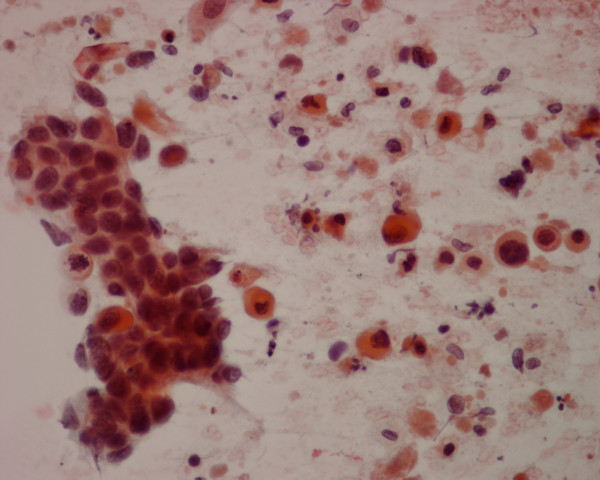
The thyroid needle aspirate in patient 1 yields a sheet of nonkeratinizing malignant squamous cells admixed with several single, keratinizing malignant squamous cells (Papanicolaou stain, × 400).

**Figure 2:**
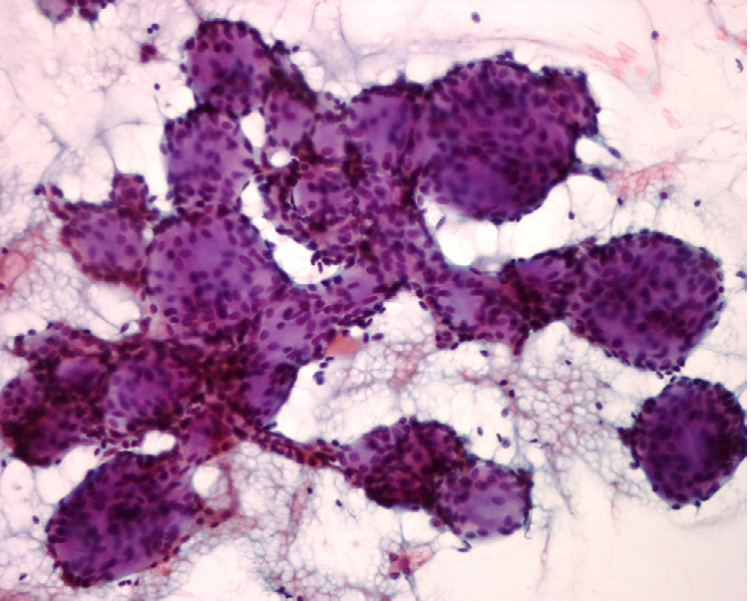
The needle aspirate in patient 2 shows small malignant cells with scant cytoplasm wrapping around basophilic, round globules, characteristic for an adenoid cystic carcinoma (Papanicolaou stain, × 250).

**Figure 3:**
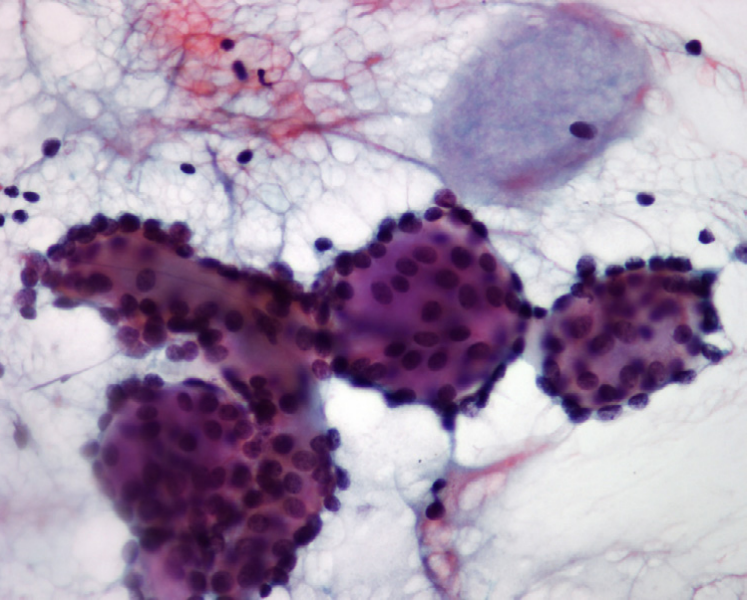
Higher magnification of a few tumor cell clusters in patient 2. A round, basophilic body is noted (Papanicolaou stain, × 400).

**Figure 4:**
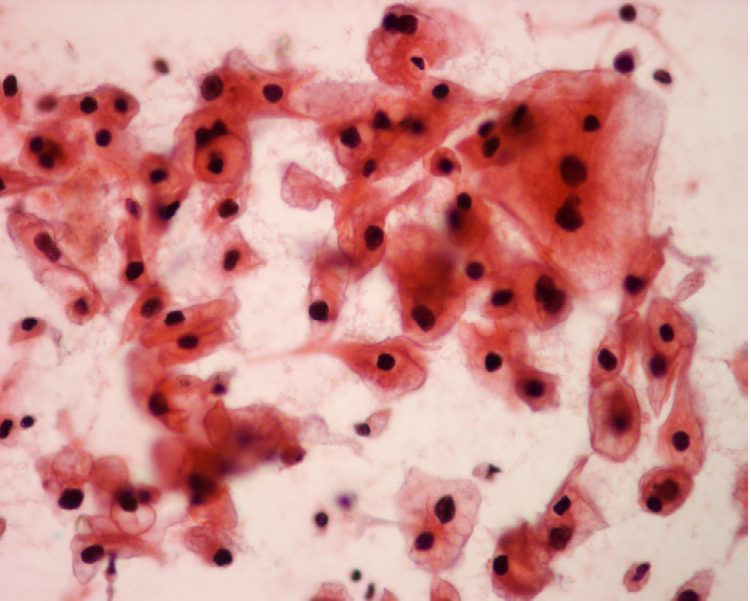
The thyroid needle aspirate in patient 3 yields monolayered sheets of malignant glandular cells with granular or clear cytoplasm, slightly pleomorphic nuclei and conspicuous nucleoli, suggesting a metastatic renal cell carcinoma, clear cell type (Papanicolaou stain, × 400).

**Figure 5:**
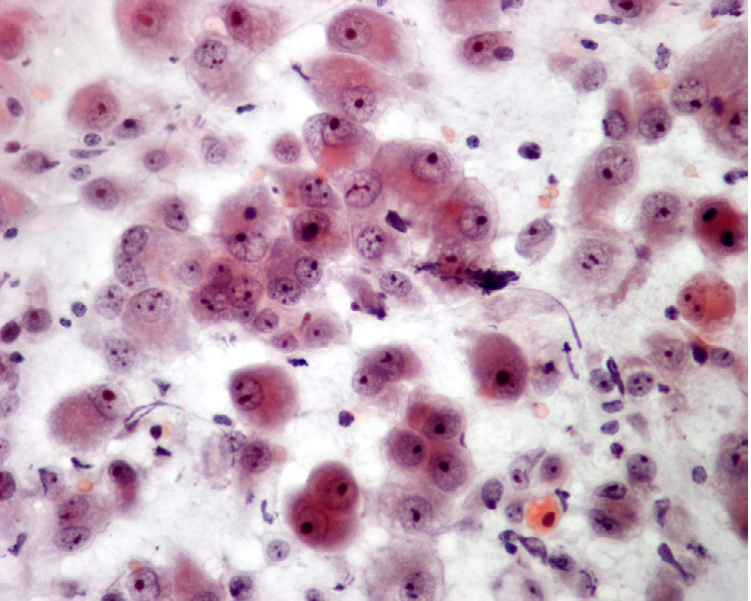
The thyroid needle aspirate in patient 4 shows single and loosely clustered polygonal cells with abundant, granular cytoplasm and eccentrically located oval nuclei with prominent nucleoli, suggesting a Hurthle cell carcinoma versus a metastatic amelanotic melanoma (Papanicolaou stain, × 400).

**Figure 6:**
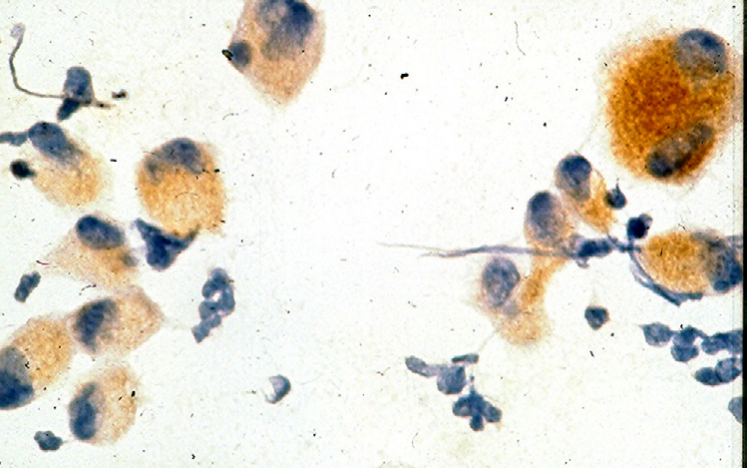
Staining of the aspirated tumor cells from patient 4 with HMB-45 antibody shows strong positive cytoplasmic reaction with this antibody, indicating a metastatic melanoma to his thyroid (avidin-biotin-complex technique, × 400).

In all five cases, the thyroid needle aspirates revealed abundant malignant cells admixed with a small number of benign follicular epithelial cells. Sheets of non-keratinizing malignant squamous cells admixed with isolated keratinizing malignant squamous cells were present in the thyroid needle aspirate of patient 1, indicating a moderately differentiated squamous cell carcinoma (Figure 1). The thyroid tumor in this patient was most likely a metastatic neoplasm, as the cytologic finding in his thyroid FNA was similar to that of the needle aspirate of his previously resected bronchogenic squamous cell carcinoma. Patient 2 showed in her thyroid FNA small malignant cells with scant cytoplasm and small, oval hyperchromatic nuclei wrapping around round basophilic globules, characteristic for a metastatic adenoid cystic carcinoma. A few round basophilic bodies were also present (Figures 2 and 3). The cytologic findings in this patient were similar to those of the needle aspirate from her previously resected parotid adenoid cystic carcinoma. In patient 3 with a previously resected renal cell carcinoma (RCC), the thyroid FNA revealed irregular large, monolayered sheets of malignant epithelial cells with granular and clear cytoplasm, suggesting a metastatic RCC, clear cell type (Figure 4). However, a thyroid Hurthle cell carcinoma was not ruled out with certainty. The tumor cells in this patient stained negatively with thyroglobulin antibody and further confirmed that the patient's lesion was a metastatic RCC. Patient 4 who had a previously resected scalp melanoma yielded in his thyroid FNA revealed several single and loosely clustered malignant large polygonal cells with oval nuclei, conspicuous nucleoli and abundant, granular cytoplasm without intracytoplasmic melanin pigment granules, suggesting a metastatic amelanotic melanoma (Figure 5). However, a Hurthle cell or anaplastic carcinoma of the thyroid was not ruled out with confidence on cytologic basis alone. Two cellular smears from this patient were stained with thyroglobulin and HMB-45 antibodies. The tumor cell cytoplasm reacted negatively with thyroglobulin and positively with HMB-45 antibodies (Figure 6). The immunocytochemical results in this case indicated a metastatic amelanotic melanoma to the thyroid. In patient 5, the thyroid FNA revealed large, monolayered sheets of malignant epithelial cells with granular or clear cytoplasm that were similar to those of patient 2, and a metastatic RCC, clear cell type to her thyroid was suggested. However, neither a Hurthle cell tumor nor a carcinoma of the thyroid with clear cell change was not ruled out. A Papanicolaou-stained cellular smear from this patient was stained with thyroglobulin antibody. The tumor cell cytoplasm reacted negatively with this antibody, suggesting a metastatic RCC, clear cell type. Further clinical and diagnostic imaging studies in patient 5 revealed a 7-cm tumor in her right kidney that was subsequently proven to be a clear cell RCC by histologic examination of her resected renal kidney.

### Histologic findings

**Figure 7:**
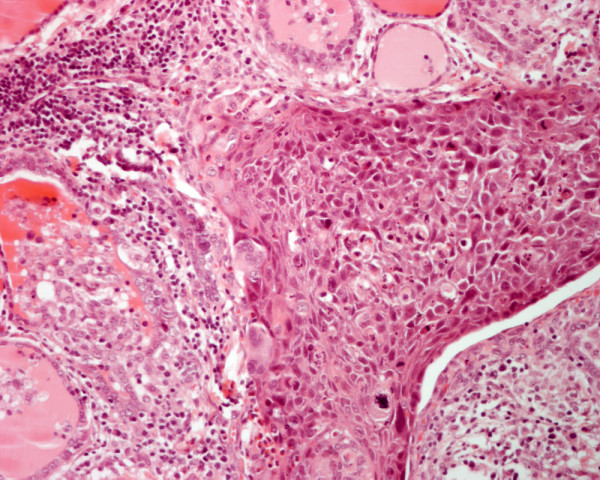
Histology of a moderately differentiated squamous cell carcinoma metastatic to the thyroid in patient 1. Residual thyroid follicles are present elsewhere (hematoxylin and eosin, × 250).

**Figure 8:**
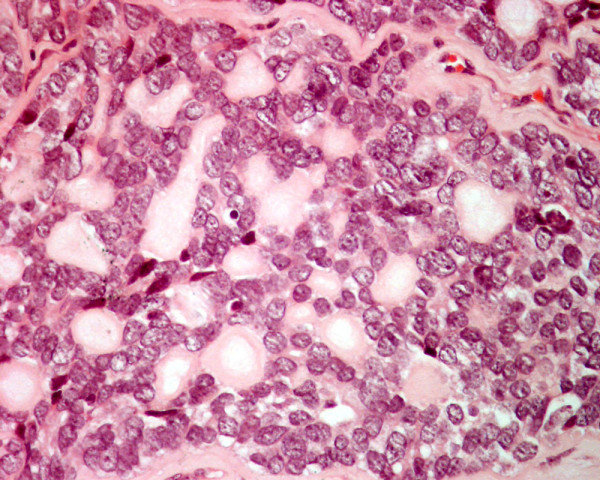
Histology of an adenoid cystic carcinoma metastatic to the thyroid in patient 2 shows small cells surrounding round spaces containing pale, slightly basophilic material (hematoxylin and eosin, × 250).

**Figure 9:**
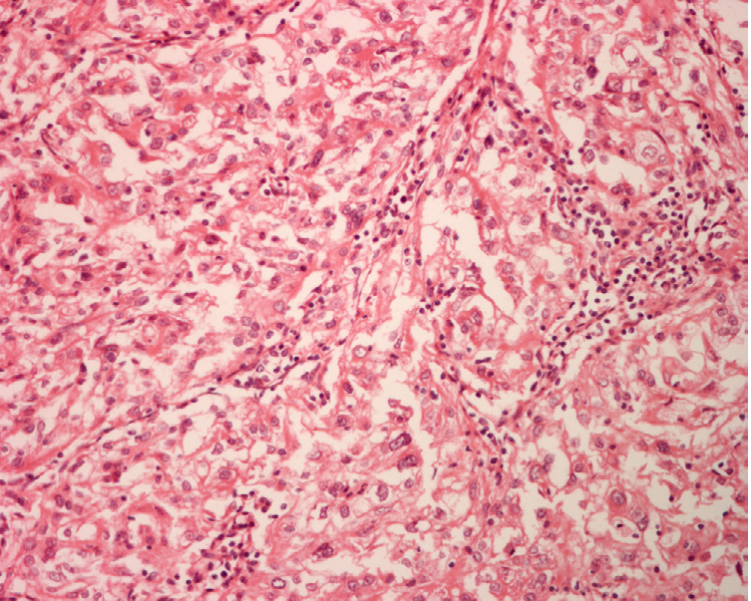
Histology of a renal cell carcinoma, clear cell type, metastatic to the thyroid in patient 3 shows tumor cells with granular cytoplasm, oval nuclei and conspicuous nucleoli arranged in glandular pattern (hematoxylin and eosin, × 250).

**Figure 10:**
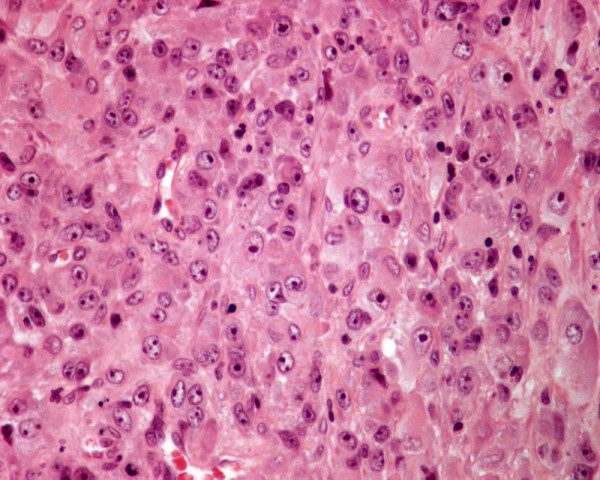
Histology of metastatic amelanotic melanoma to the thyroid in patient 4 shows large malignant polygonal cells with oval nuclei and prominent nucleoli, in solid pattern (hematoxylin and eosin, × 250).

The primary tumors and the thyroid tumors in patients 1 to 4 were histologically similar and showed histologic features of a moderately differentiated bronchogenic squamous cell carcinoma, a parotid adenoid cystic carcinoma, an RCC and a cutaneous melanoma metastatic to the thyroid (Figures 7, 8, 9, 10). In patient 5 the thyroid tumor was histologically similar to the RCC of his right kidney, indicating a metastatic RCC to his thyroid.

## Discussion

In an analysis of 63 patients with nonthyroidal cancer, 45 (71%) of thyroid nodular lesions were benign, 11 (17%) were metastases, 4 (6%) were primary thyroid carcinomas, 1 was Hashimoto's thyroiditis and 2 were inconclusive [6]. All types of solid cancer arising from different anatomic sites can metastasize to the thyroid, with kidney being the most common primary tumor site (33%), followed by lung (16%), breast (16%), esophagus (9%) and uterus (7%) [7]. These metastatic cancers to the thyroid can be diagnosed cytologically by FNA and managed appropriately [7,8].

Metastatic cancers in the thyroid accounted for 0.1% of all thyroid nodular lesions that were investigated by FNA, according to one large cytology series consisting of about 25,000 cases [9]. From the cytodiagnostic point of view, diagnosis of a metastatic cancer to the thyroid, in the majority of cases, is relatively straightforward, as the aspirated tumor cells are cytologically similar to those of the primary tumor and different from those of the usual thyroid carcinomas [7-10]. In our first reported case, the thyroid FNA revealed malignant squamous cells which were most likely derived from a metastatic squamous cell carcinoma to the thyroid rather than from a primary squamous cell carcinoma of the gland which is a very rare tumor [10]. In the second patient, although the cytologic findings in her thyroid FNA were characteristic for an adenoid cystic carcinoma [12], a microfollicular thyroid carcinoma may sometime display a similar cytologic pattern, however [13]. In the third case monolayered sheets of malignant cells with granular and clear cytoplasm were most likely in keeping with a metastatic clear cell RCC [14]. However, a primary thyroid carcinoma with clear cell change [15] may show similar cytologic findings in FNA. In patient 4, the presence of large malignant cells with granular cytoplasm in his thyroid FNA would indicate a metastatic amelanotic melanoma, a Hurthle cell carcinoma or an anaplastic carcinoma of the thyroid. Immunocytochemical study with HMB-45 and thyroglobulin antibodies proved to be necessary to confirm a metastatic melanoma and rule out a thyroid carcinoma. Cytodiagnosis of metastatic adenocarcinoma to the thyroid by FNA can be challenging as metastatic RCC and mammary adenocarcinoma may yield cells readily mistaken for those of a follicular or papillary carcinoma of the thyroid [9]. Immunocytochemical staining of the aspirated tumor cells with thyroglobulin is useful for tumor typing as cells derived from a thyroid follicular tumor commonly express thyroglobulin. Cells of an RCC are rich in glycogen and lipid. A positive reaction of the tumor cell cytoplasm with periodic acid-Schiff without prior diastase digestion and with Oil-red-O will further support the diagnosis of metastatic RCC [5].

Metastatic cancer to the thyroid usually occurs in patients with disseminated cancers and is associated with a dismal prognosis, with death occurring in 9 months on average [1,2]. Patients with solitary thyroid metastatic RCC may have a prolonged survival after surgical resection and adjuvant therapy [4,5]. Metastatic RCC to the thyroid deserves some comments. In one large series consisting of 36 cases of metastatic RCC to thyroid, 23 patients (64%) had previous evidence of an RCC as remote as 21.8 years before the thyroid metastases (mean, 9.4 years), and the metastatic tumor to the thyroid was the initial manifestation of RCC in 13 patients [5]. The majority of patients (n = 23; 64%) died with disseminated disease (mean, 4.9 years) but 13 patients (36%) were alive or had died without evidence of disease (mean, 9.1 years). In our report, all 5 patients died with metastatic disease from 27 to 40 months after their thyroid surgeries, and the 2 patients with solitary metastatic RCC had longest survival periods of 38 and 40 months, respectively.
